# TMED2/9/10 Serve as Biomarkers for Poor Prognosis in Head and Neck Squamous Carcinoma

**DOI:** 10.3389/fgene.2022.895281

**Published:** 2022-06-08

**Authors:** Wen Gao, Zhe-Wen Zhang, Hong-Yi Wang, Xin-Di Li, Wei-Ting Peng, Hao-Yu Guan, Yu-Xuan Liao, An Liu

**Affiliations:** ^1^ Department of Otolaryngology-Head and Neck Surgery, Third Xiangya Hospital, Central South University, Changsha, China; ^2^ Xiangya School of Medicine, Central South University, Changsha, China; ^3^ Graduate School of Peking Union Medical College, Chinese Academy of Medical Sciences and Peking Union Medical College, Beijing, China

**Keywords:** head and neck squamous carcinoma, TMED, biomarkers, prognosis, bioinformatics analysis

## Abstract

**Background:** Head and neck squamous carcinoma (HNSC) is one of the most common malignant tumors with high incidence and poor prognosis. Transmembrane emp24 structural domain (TMED) proteins are involved in protein transport and vesicle budding processes, which have implicated various malignancies’ progression. However, the roles of TMEDs in HNSC, especially in terms of development and prognosis, have not been fully elucidated.

**Methods:** We applied TIMER 2.0, UALCAN, GEPIA 2, Kaplan-Meier plotter, GEO, The Human Protein Atlas (HPA), cBioPortal, Linkedomics, Metascape, GRNdb, STRING, and Cytoscape to investigate the roles of TMED family members in HNSC.

**Results:** Compared with normal tissues, the mRNA expression levels of TMED1/2/4/5/7/8/9/10 were significantly increased in the TCGA HNSC dataset. And we combined GEPIA 2 and Kaplan-Meier Plotter to select TMED2/9/10 with prognostic value. Then we detected the levels of mRNA in the GEO HNSC database and the protein expression in HPA. It was found that the mRNA and protein expression levels of TMED2/9/10 were increased in HNSC. Gene Ontology (GO) and Kyoto Encyclopedia of Genes and Genomes (KEGG) analysis showed that TMED2/9/10 and their co-expressed genes promoted the malignant behavior of tumors by participating in biological processes such as intracellular transferase complex, protein transport, focal adhesion, intracellular protein processing. Single-cell analysis and immune infiltration analysis suggested that immune responses of cancer-associated fibroblasts and endothelial cells might be associated with prognosis. Finally, the transcription factors-genes network and protein-protein functional interaction network pointed to genes such as X-box binding protein 1 (XBP1) and TMED7, which might cooperate with TMED2/9/10 to change the progression of HNSC.

**Conclusions:** Our study implied that TMED2/9/10 and related genes mightjointly affect the prognosis of HNSC, providing specific clues for further experimental research, personalized diagnosis strategies, and targeted clinical therapy for HNSC.

## Introduction

Head and Neck Squamous Carcinoma (HNSC) is the most common head and neck region malignancy, mainly from the mucosal epithelium of the oral cavity, pharynx, and larynx (Bhat et al., 2021). Unfortunately, HNSC patients were usually diagnosed at an advanced stage due to the small size of HNSC lesions and the lack of effective indicators for early detection of tumor development. Therefore, this carcinoma currently has a 5-year survival rate less than 65% (Miller et al., 2016). At the same time, not only the characteristics of HNSC prone to recurrence and metastasis but also the dramatic decrease in the quality of life of patients seriously threatens the overall survival (Osazuwa-Peters et al., 2018; Saada-Bouzid et al., 2019). Therefore, we urgently need to develop new biomarkers for early screening and diagnosis to improve patient prognosis.

Transmembrane emp24 structural domain (TMED) proteins, also known as p24 proteins, are associated with bidirectional transport processes between the endoplasmic reticulum and the Golgi apparatus. According to previous studies, abnormal expression of TMED proteins with related pathways was closely associated with poor prognosis in many diseases, such as non-alcoholic fatty liver, multiple myeloma, diabetes, Alzheimer’s disease, strong chordoma, osteoarthritis (Wang et al., 2012; Hou et al., 2017; Shin et al., 2019; Ge et al., 2020; Yang J. et al., 2021; Huang et al., 2021). For instance, TMED2 was expressed higher in sphere-shaped clones (SCs) and might play a role in cancer cell proliferation; the increased expression of TMED2 was significantly related to unfavorable outcomes in patients with breast cancer (Sial et al., 2021). TMED3 played a role in promoting the progression and development of lung squamous cell carcinoma, liver cancer, and breast progression (Zheng et al., 2016; Pei et al., 2019; Xie et al., 2021), and TMED8 methylation was a novel predictive and prognostic feature for patients with high-risk neuroblastoma (Liu and Li, 2021). Besides, the high expression of TMED9 might promote the proliferation of cancer cells by inhibiting autophagy and predict poor prognosis in hepatocellular carcinoma (HCC) and colon cancer (Schwarz and Allikmets, 2019; Ju et al., 2021). In addition, downregulated Golgi-endoplasmic reticulum (ER) traffic mediators TMED2 and TMED10 were related to positive prognosis in Prostatic cancer (PCa) (Chen and Hu, 2019). Therefore, TMED proteins might serve as prognostic markers to predict tumor prognosis. Current studies have found that the expression level of TMED2 in HNSC was up-regulated and related to different cancer stages, races, genders, and ages (Sial et al., 2021). Nevertheless, the potential prognosis value of the TMED family has not been fully elucidated in HNSC.

In this study, we first examined the expression level of the TMED family in HNSC tissues and their prognostic value. With the above analyses, we identified TMED2/9/10 as diagnosis and prognosis biomarkers for HNSC. Further, we performed expression-related gene analysis, GO and KEGG enrichment analysis, single-cell analysis, and immune infiltration analysis of TMED2/9/10 to elaborate on their physiological and immune functions. Based on the functional interaction of TMED proteins, we discovered other potential prognostic molecular biomarkers and validated the role of these genes in HNSC progression. Our experimental results may provide research directions for future studies of molecular biomarkers of HNSC development and prognosis, leading to new diagnosis and treatment modalities based on risk stratification.

## Materials and Methods

### TIMER 2.0

TIMER 2.0 (http://timer.cistrome.org/) is The Cancer Genome Atlas (TCGA) database visual portal for the analysis of gene expression differences between tumor and normal tissues and the association between gene expression and immune infiltration (Li et al., 2020). We used the “Gene_DE” module in TIMER 2.0 to analyze the differential TMED expression between HNSC and normal tissues. Moreover, the “Gene” module and “Correlation” module was used to obtain correlation analysis between TMED2/9/10 and immune cell infiltration levels in HNSC (Immune Infiltrates: Cancer-associated fibroblasts, Endothelial cells, B cells). These analyses were performed using the TCGA HNSC dataset (*n* = 520) by spearman analysis, and differences with a *p*-value < 0.05 were considered statistically significant. The gene expression levels were displayed with log2 RSEM.

### UALCAN

UALCAN (http://ualcan.path.uab.edu/index.html) is a comprehensive web tool based on TCGA database (Chandrashekar et al., 2017). “TCGA Gene analysis” module was used to analyze mRNA levels of the TMED2/9/10 in HNSC patients and healthy individuals and their correlation with clinicopathological parameters, including age, gender, tumor grade, lymph node metastasis, TP53 mutation status, and cancer stage. These analyses were performed using the TCGA HNSC dataset (*n* = 520), with *p*-values < 0.05 considered statistically significant results.

### Kaplan-Meier Plotter

Kaplan-Meier Plotter (http://kmplot.com/analysis) was used to analyze the correlation between the mRNA expression of the TMED family and overall survival in HNSC patients (Nagy et al., 2021). We can perform pan-cancer analysis by selecting the “Pan-cancer RNA-seq” module. According to high versus low expression, the patient sample (*n* = 499) was divided into two groups. The result was assessed by Kaplan-Meier overall survival charts, expressed as risk ratios, 95% confidence intervals, and calculated log-rank *p*-value.

### GEPIA 2

GEPIA 2 (http://gepia2.cancer-pku.cn/) provides an in-depth analysis of gene expression data based on TCGA and Genotype-Tissue Expression (GTEx) data (Tang et al., 2019). This study used the “Survival Analysis” module to analyze the relationship between TMED genes and Overall Survival (OS) in HNSC patients. The relevant parameters were set as follows: Group Cutoff = Median, add Hazards Ratio (HR) and 95% Confidence Interval, Axis Units = Months. Moreover, we used the “Similar Genes Detection” module to explore the top 1000 genes that have related expression patterns with TMED2/9/10.

### The Human Protein Atlas

The Human Protein Atlas (HPA, https://www.proteinatlas.org/) is an online database that represents protein expression by immunohistochemical staining techniques (Uhlen et al., 2017). We compared TMED2/9/10 protein expression levels in normal and tumor tissues by using the “TISSUE” and “PATHOLOGY” modules. The protein expression scores were based on manually scored immunohistochemical data, including staining intensity (Not detected, Low, Medium or High). The following tissue information was used in this study: patient ID: 2615, male, 17 years old, tonsil (T-61100), normal tissue, NOS (M-00100); patient ID: 2513, male, 27 years old, tonsil (T-61100), normal tissue, NOS (M-00100); patient ID: 2608, male, 51 years old, skeletal muscle (T-13000), head and neck (T-Y0000), squamous cell carcinoma, NOS (M-80703).

### cBioPortal

cBioPortal (https://www.cbioPortal.org/) is a repository of cancer genomics datasets from the TCGA database for genomics analysis (Gao et al., 2013). Based on the TCGA HNSC dataset (Nature 2015, 279 total samples), the “Query” module was analyzed for mRNA levels of TMED2/9/10 with Genomic Profiles set to Mutations, Structural Variant, Putative copy-number alterations from GISTIC and mRNA expression Z-score relative to all samples (log RNA Seq V2 RSEM). The case set is complete samples (279). Mutation data were obtained from whole-exome sequencing. The mutation rate of TMED2/9/10 in HNSC compared to normal tissues and expression Heatmap of TMED2/9/10 was detected.

### LinkedOmics

LinkedOmics (http://www.linkedomics.org/) is a TCGA database visual web portal for genomics analysis (Vasaikar et al., 2018). The LinkedOmics database was used to identify TMED2/9/10 co-expressed genes, and the number of positive/negative genes was counted separately. We used the Pearson correlation coefficient to analyze the TMED2/9/10 data (*n* = 517) from the RNAseq of TCGA (HNSC), resulting in 20163 related genes.

### Metascape

Metascape (http://metascape.org/) is an open database for studying the functions between genes of interest, using the GO and KEGG databases for pathway enrichment analysis (Zhou et al., 2019). We used Metascape to perform pathway enrichment analysis of TMED2/9/10 and co-expressed genes. Studies were carried out with the default parameters of minimal overlap = 3, minimal enrichment = 3, and *p*-value cutoff = 0.01.

### GRNdb

GRNdb (http://www.grndb.com/) is a gene regulatory network database that provides a reliable way to predict transcription factors associated with genes (Fang et al., 2021). In this study, the “Exact Search” module was used to reveal the upstream regulatory transcription factors of TMED2/9/10 and hub genes in HNSC, as well as to explore the expression levels of TMED2/9/10 and hub genes in different cells. The NES (Normalized Enrichment Score for TF-target pair) value = ALL.

### Search Tool for the Retrieval of Interacting Genes and Cytoscape

The STRING database (http://string-db.org/) is an accessible online database to predict PPI information with parameters set to Network Type = physical subnetwork, Required score = 0.900, Size cutoff = no more than ten interactions (Szklarczyk et al., 2021). STRING drew a protein network to discover the interactions between TMED2/9/10 and other proteins, and the results were visualized in Cytoscape software. The obtained PPI network was analyzed by cytoHubba plugin with parameters set to Hubba nodes = Top 10 nodes ranked by degree. (Version: Cytoscape_v3.9.0) (Shannon et al., 2003; Chin et al., 2014).

### Microarray Data

The Gene Expression Omnibus (GEO) database (http://www.ncbi.nlm.nih.gov/geo/) is an online gene expression database containing high-throughput microarray and next-generation sequence functional genomic datasets (Barrett et al., 2013). Two HNSC datasets (GSE13601 and GSE89923) were retrieved and downloaded from the GEO database. GSE13601 contains gene expression profiles of patients with oral tongue squamous cell carcinoma (*n* = 37) and patients with normal mucosa (*n* = 20); Platforms: Affymetrix Human Genome U95 Version 2 Array (Estilo et al., 2009). GSE89923 contains gene expression profiles of patients with oral squamous cell carcinoma (*n* = 57) and normal human gingival epithelial cells (*n* = 33); Platforms: Affymetrix Human Genome U95 Version 2 Array (Woo et al., 2017).

### Statistical Analysis

GEO dataset was downloaded using R language GEOquery package as an external validation (Davis and Meltzer, 2007), and the data was normalized by Limma package “normalizeBetweenArrays” function to obtain the expression of TMED2/9/10 in normal head and neck tissues and HNSC (Bolstad et al., 2003; Ritchie et al., 2015). The rank-sum test was used for this analysis. The statistical analysis of the survival data was completed with the survivor R package, and the visualization was carried out with the survminer R package. The correlation analysis was done by using the Spearman method. “ggplot2” package of R software (Version:3.3.3) was used for data visualization (Maag, 2018).

## Results

### Defining the TMED Family in HNSC

**FIGURE 1:**
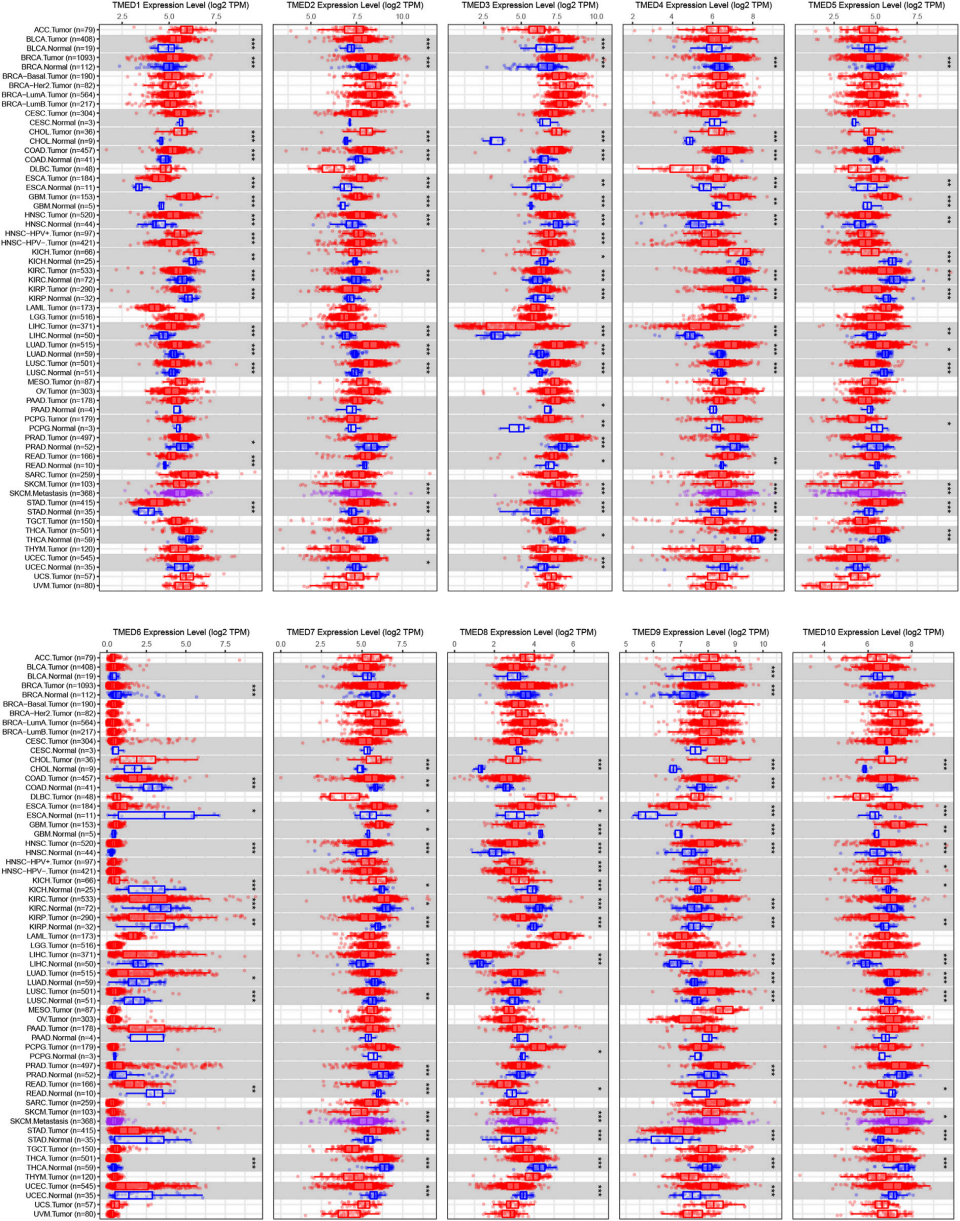
Expression levels of the TMED family in different types of tumor tissues and normal tissues from the TIMER 2.0 database. (* *p*-value < 0.05, ** *p*-value < 0.01, *** *p*-value < 0.001).

**FIGURE 2:**
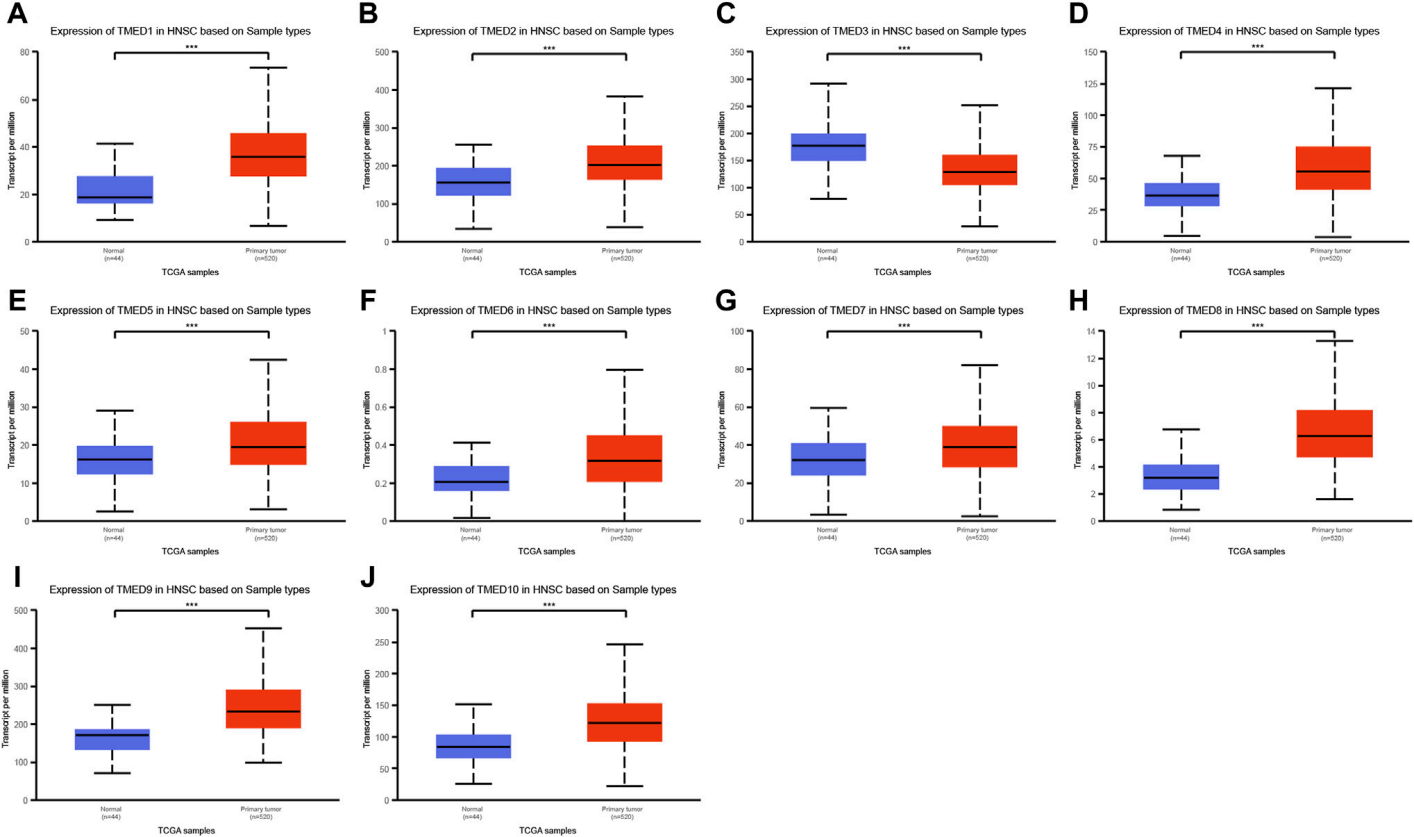
Expression levels of the TMED family in HNSC patients based on UALCAN database. **(A)** TMED1; **(B)** TMED2; **(C)** TMED3; **(D)** TMED4; **(E)** TMED5; **(F)** TMED6; **(G)** TMED7; **(H)** TMED8; **(I)** TMED9; **(J)** TMED10. (***, *p*-value < 0.001)

The TIMER 2.0 database was used to analyze 10 genes in the TMED family and to assess the expression levels of each gene in HNSC tissues and normal tissues (^∗^ *p*-value < 0.05, ^∗∗^ *p*-value < 0.01, ^∗∗∗^ *p*-value < 0.001). The results showed that TMED3 expression was down-regulated in HNSC tissues and the expression level of TMED6 was extremely low both in HNSC tissues and normal tissues. Nevertheless, the expression of the other eight genes in HNSC tissues was elevated significantly higher than normal tissues **(** Figure 1 **)**. In addition, we obtained the same results from UALCAN **(** Figure 2 **)**. The *p*-value for expression of the TMED family in HNSC versus normal tissues was statistically significant in TIMER 2.0 and UALCAN (*p*-value < 0.05) **(** Table 1 **)**.

**TABLE 1:** The *p*-value for expression of the TMED family in HNSC versus normal tissues in TIMER 2.0 and UALCAN.

Name	*p*-Value in TIMER 2.0	*p*-Value in UALCAN
TMED1	<1E-12	1.62E-12
TMED2	8.35E-08	2.81E-10
TMED3	3.45E-05	8.40E-04
TMED4	3.81E-06	1.91E-06
TMED5	1.55E-03	1.41E-04
TMED6	2.67E-06	7.84E-12
TMED7	2.59E-04	1.88E-05
TMED8	<1E-12	<1E-12
TMED9	<1E-12	1.62E-12
TMED10	4.06E-08	5.05E-10

### Prognostic Value of TMED 2/9/10 in HNSC

**FIGURE 3:**
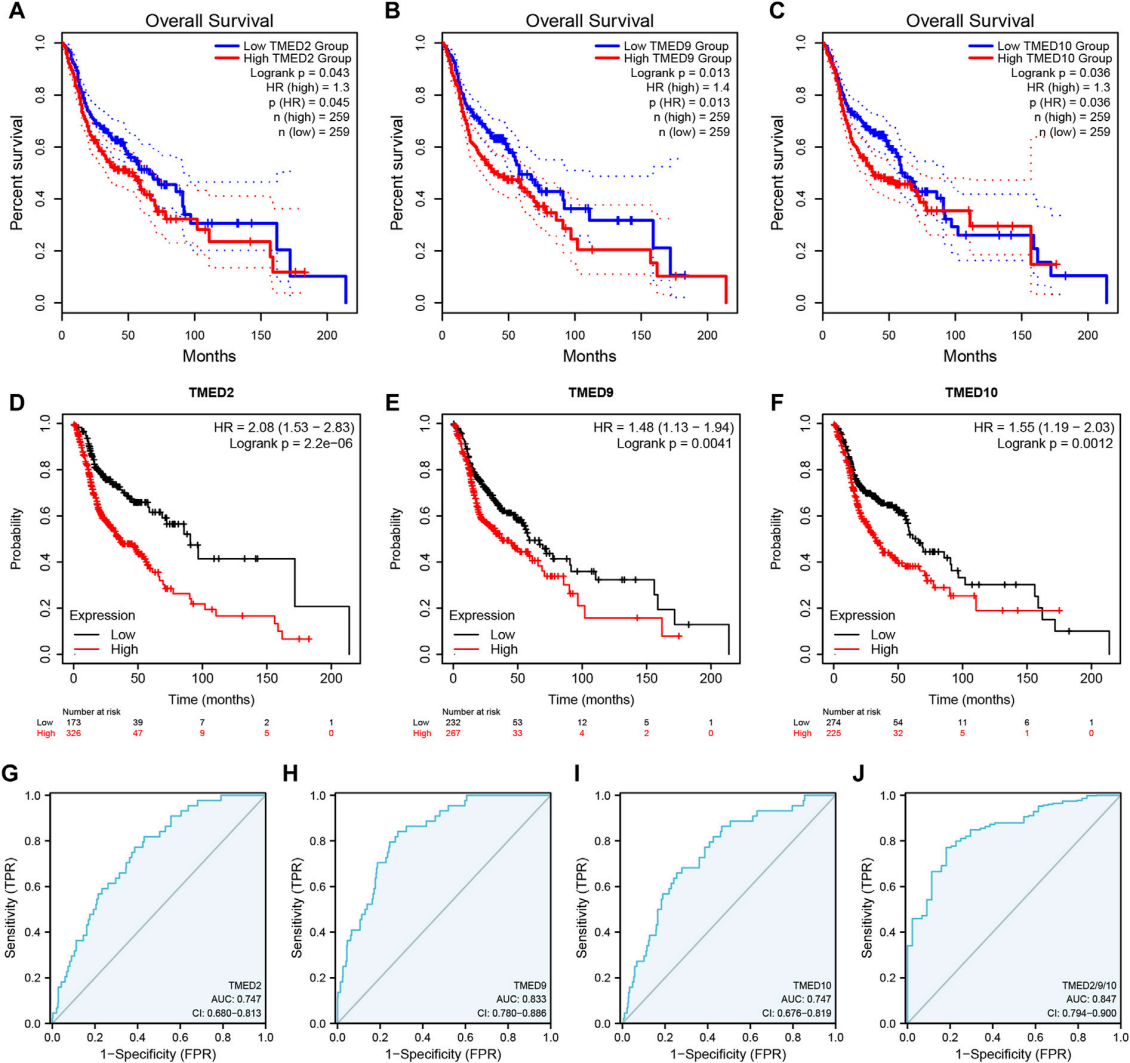
The prognostic value of TMED2/9/10 in HNSC patients based on **(A–C)** the GEPIA 2 database and **(D–F)** Kaplan-Meier Plotter database. The diagnostic value of **(G)** TMED2, **(H)** TMED9, **(I)** TMED10 and **(J)** the combination of TMED2/9/10 in HNSC patients.

To better understand the prognostic value of the TMED family in HNSC, we investigated the relationship between the TMED family expression and OS in HNSC patients through the GEPIA 2 (Supplementary Figure S1). By using the GEPIA 2, the results showed that HNSC patients with high TMED2/9/10 expression had a worse prognosis than those with low expression (*p*-value < 0.05) (Figure 3A–C), while other members of the TMED family were not statistically significant in survival analysis (Supplementary Figure S1). Therefore, we considered TMED2/9/10 as prognostic markers for HNSC. Moreover, we analyzed its survival value by performing survival curves in the Kaplan-Meier Plotter database. We also found that the higher expression levels of TMED2/9/10 were closely connected with worse prognosis, which indicated the significant prognostic value in HNSC (*p*-value < 0.05) (Figures 3D–F). Additionally, we affirmed the diagnostic value of TMED2/9/10 in HNSC patients with the help of the receiver operating characteristic curve (AUC >0.5) (Figures 3G–I). Surprisingly, when we combined three genes as a new biomarker, its diagnostic value became more significant (AUC = 0.847) (Figure 3J). The above results suggested that the expression level of TMED2/9/10 had the capacity to serve as potential diagnostic biomarkers in HNSC diagnosis.

### Further Validation of TMED2/9/10 Expression Levels

**FIGURE 4:**
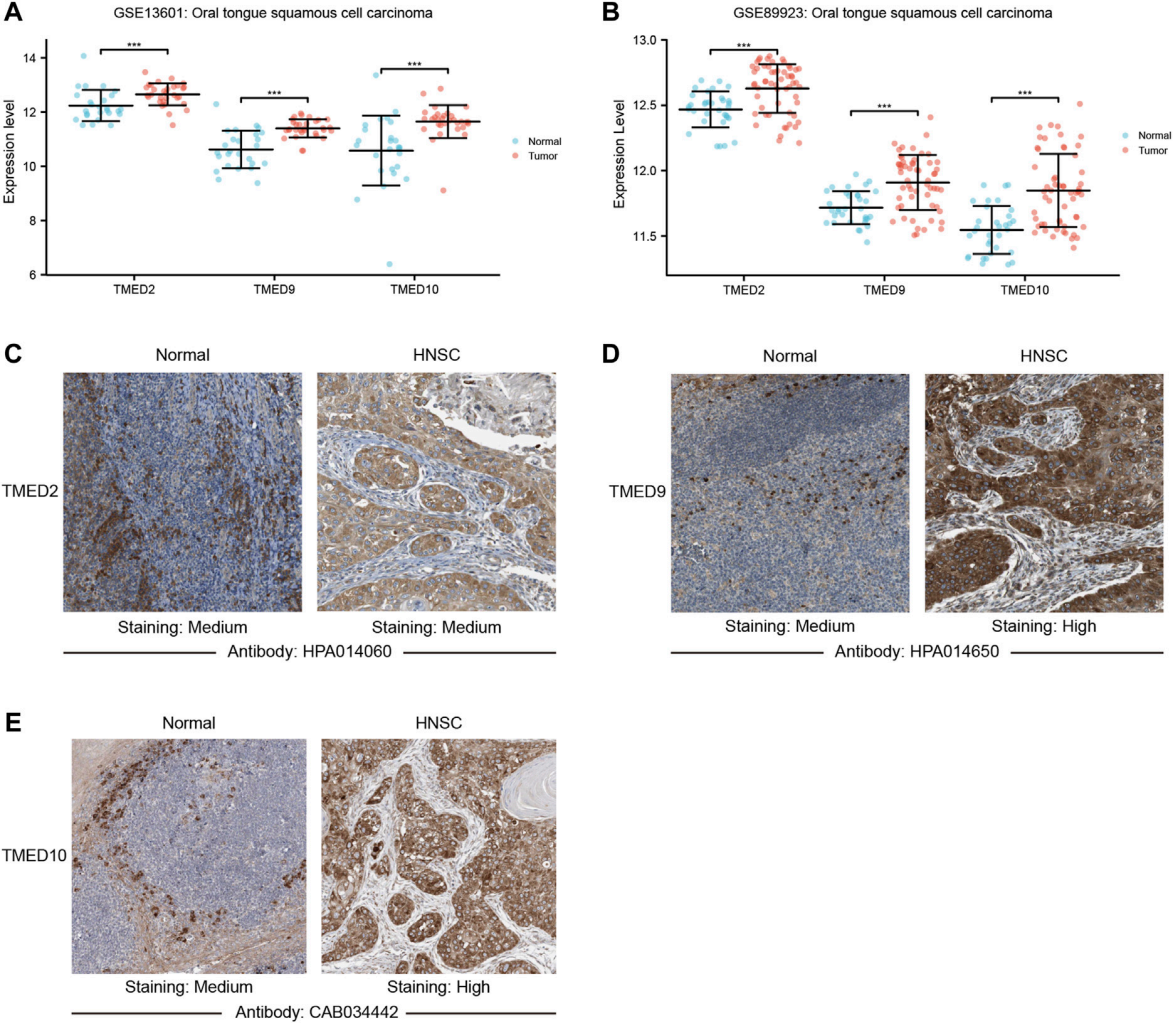
The mRNA expression levels between tumor and non-tumor tissues in head and neck squamous cell carcinoma (HNSC) patients in the GEO dataset including **(A)** GSE13601; **(B)** GSE89923 (***, *p*-value < 0.001). Protein expression levels of TMED2/9/10 in normal head and neck tissues and HNSC of The Human Protein Atlas. **(C)** TMED2; **(D)** TMED9; **(E)** TMED10.

To further validate the role of TMED2/9/10 in HNSC, we explored the mRNA expression levels by using the GEO dataset. We found that TMED2/9/10 in HNSC also showed high expression in the GEO dataset (GSE13601 and GSE89923) (Figures 4A,B). Moreover, we analyzed the protein expression levels of TMED2/9/10 by using the immunohistochemistry (IHC) data from the HPA database. The results showed that the protein expression levels of TMED9 and TMED10 were significantly different in normal head and neck tissues and HNSC, which was consistent with the above results (Figures 4D,E). However, the difference of TMED2 in normal head and neck tissues and HNSC was not significant, which may be due to data heterogeneity, resulting in the difference of protein expression levels of TMED2 from the above results (Figure 4C).

### Correlations Between the Expression Levels of TMED2/9/10 and Clinicopathological Features in HNSC

The above data indicated that TMED2/9/10 were up-regulated in HNSC tissues and had an excellent prognostic value on HNSC. Therefore, we further examined the association between TMED2/9/10 and clinicopathological features in HNSC. It was found that TMED2/9/10 were significantly associated with age, gender, cancer grade, TP53 mutation status, lymphatic metastasis, and cancer stage from UALCAN (Table 2).

**TABLE 2:** The relationships between TMED2/9/10 expression and clinicopathological features of HNSC patients in UALCAN.

Clinicopathologic features	TMED2 (*p*-value)	TMED9 (*p*-value)	TMED10 (*p*-value)
Age			
Normal-vs-Age (21–40Yrs)	1.15E-04	4.48E-05	5.19E-04
Normal-vs-Age (41–60Yrs)	4.69E-09	1.62E-12	3.64E-09
Normal-vs-Age (61–80Yrs)	8.07E-10	<1E-12	2.64E-09
Normal-vs-Age (81–100Yrs)	1.94E-03	4.81E-04	6.68E-03
Gender			
Normal-vs-Male	2.73E-10	1.62E-12	1.38E-09
Normal-vs-Female	6.59E-08	4.25E-14	1.57E-07
Cancer stage			
Normal-vs-Stage1	4.06E-03	5.35E-04	6.42E-05
Normal-vs-Stage2	1.42E-07	1.44E-11	2.22E-04
Normal-vs-Stage3	1.58E-05	1.11E-10	2.23E-06
Normal-vs-Stage4	4.71E-10	1.62E-12	4.68E-10
Cancer grade			
Normal-vs-Grade 1	1.37E-02	7.41E-08	2.97E-02
Normal-vs-Grade 2	1.44E-10	1.62E-12	1.51E-11
Normal-vs-Grade 3	4.94E-08	1.62E-12	1.20E-07
Normal-vs-Grade 4	1.88E-03	5.24E-03	2.17E-02
Grade 1-vs-Grade 2	3.92E-03	NS	6.94E-07
Grade 1-vs-Grade 3	6.72E-03	6.96E-03	3.78E-03
Grade 2-vs-Grade 3	NS	3.16E-02	4.91E-02
Lymphatic metastasis			
Normal-vs-N0	2.11E-08	1.63E-12	4.53E-08
Normal-vs-N1	8.16E-05	1.28E-10	1.45E-04
Normal-vs-N2	NS	1.00E-03	1.46E-02
Normal-vs-N3	9.84E-03	8.59E-07	NS
N0-vs-N2	NS	4.50E-02	NS
TP53 mutation			
Normal-vs-TP53-Mutant	5.88E-12	1.62E-12	3.65E-12
Normal-vs-TP53-NonMutant	3.52E-05	1.63E-12	1.62E-04
TP53-Mutant-vs-TP53-NonMutant	4.27E-05	9.96E-03	5.33E-07

### Co-Expression and Genetic Alteration of TMED2/9/10 in HNSC.

**FIGURE 5:**
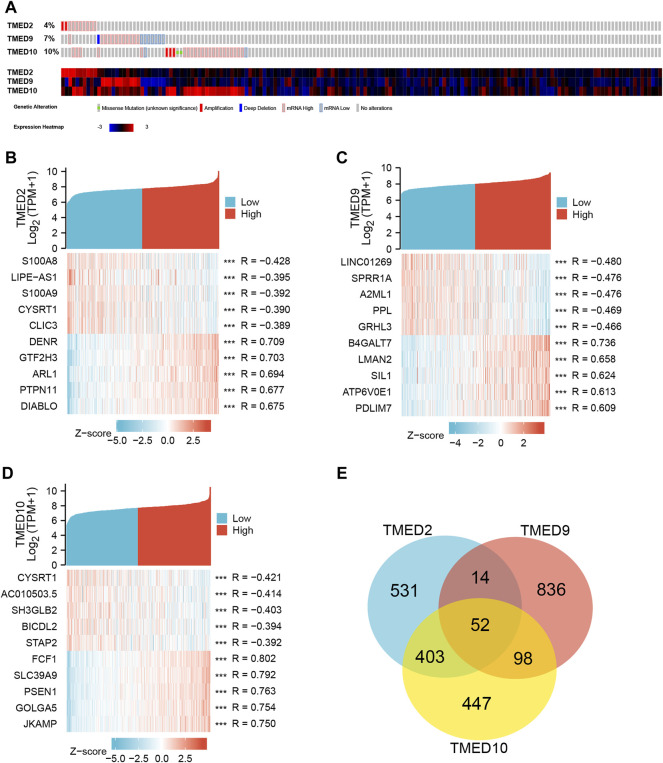
**(A)** Expression levels of TMED2/9/10 in cBioPortal database in HNSC. **(B–D)** Heatmap analysis of genes associated with TMED2/9/10 expression. **(E)** Intersection co-expression genes of TMED2/9/10.

From the CbioPortal database, the results displayed that the mRNA change rates of TMED2/9/10 in HNSC were 4%, 7%, and 10%, respectively (Figure 5A). To better interrogate the relationship between TMED2/9/10 and HNSC, we explored co-expressed genes related to TMED2/9/10 using data from TCGA HNSC patients. Among them, TMED2 had 10,154 positively correlated genes and 10,009 negatively correlated genes, TMED9 had 9,508 positively correlated genes and 10,655 negatively correlated genes, and TMED10 had 9,712 positively correlated genes and 10,451 negatively correlated genes. Five significant genes positively correlated with TMED2/9/10 and five significant genes negatively correlated with TMED2/9/10 were shown in the form of Heatmaps, respectively (Figures 5B–D). In addition, Venn diagrams indicated 52 genes co-expressed by TMED2/9/10 (Figure 5E).

### Enrichment Analysis of TMED2/9/10 in HNSC

**FIGURE 6:**
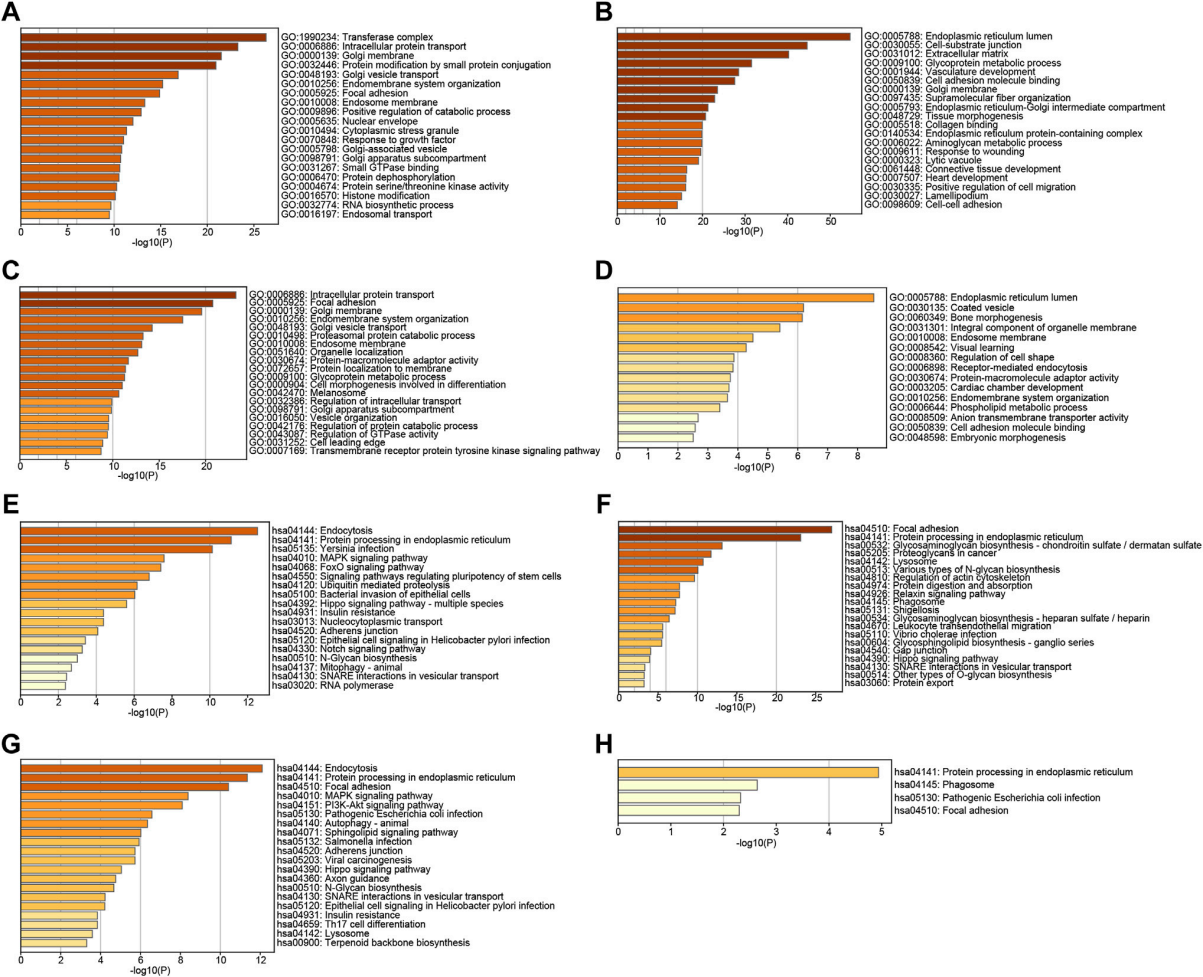
GO analysis of **(A)** TMED2, **(B)** TMED9, **(C)** TMED10 and **(D)** co-expression genes. KEGG pathway enrichment analysis of **(E)** TMED2, **(F)** TMED9, **(G)** TMED10, and **(H)** co-expression genes.

To further explore the function of TMED2/9/10 in HNSC, we used GO and KEGG analysis on TMED2/9/10 and co-expressed genes by the Metascape. GO function annotation results showed that TMED2 was mainly involved in transferase complex intracellular, protein transport, Golgi membrane, protein modification by small protein conjugation (Figure 6A); TMED9 was mainly involved in endoplasmic reticulum lumen, cell-substrate junction, extracellular matrix (Figure 6B); TMED10 was mainly involved in intracellular protein transport, focal adhesion, Golgi membrane (Figure 6C); Co-expressed genes were mainly involved in endoplasmic reticulum lumen, envelope vesicles, and bone morphogenesis (Figure 6D). KEGG pathway analysis indicated that TMED2 was enriched in regulation of endocytosis, protein processing in the endoplasmic reticulum, and Yersinia infection pathway (Figure 6E); TMED9 was enriched in focal adhesion, protein processing in the endoplasmic reticulum protein processing in the cell, focal adhesion, and protein processing in the endoplasmic reticulum (Figure 6F); TMED10 was enriched in intracellular protein processing in the endoplasmic reticulum, focal adhesion (Figure 6G); co-expressed genes in the protein processed in the endoplasmic reticulum, phagosome, pathogenic *Escherichia coli* infection, and focal adhesion (Figure 6H).

### Gene TMED/2/9/10 Expression Profiling in HNSC

**FIGURE 7:**
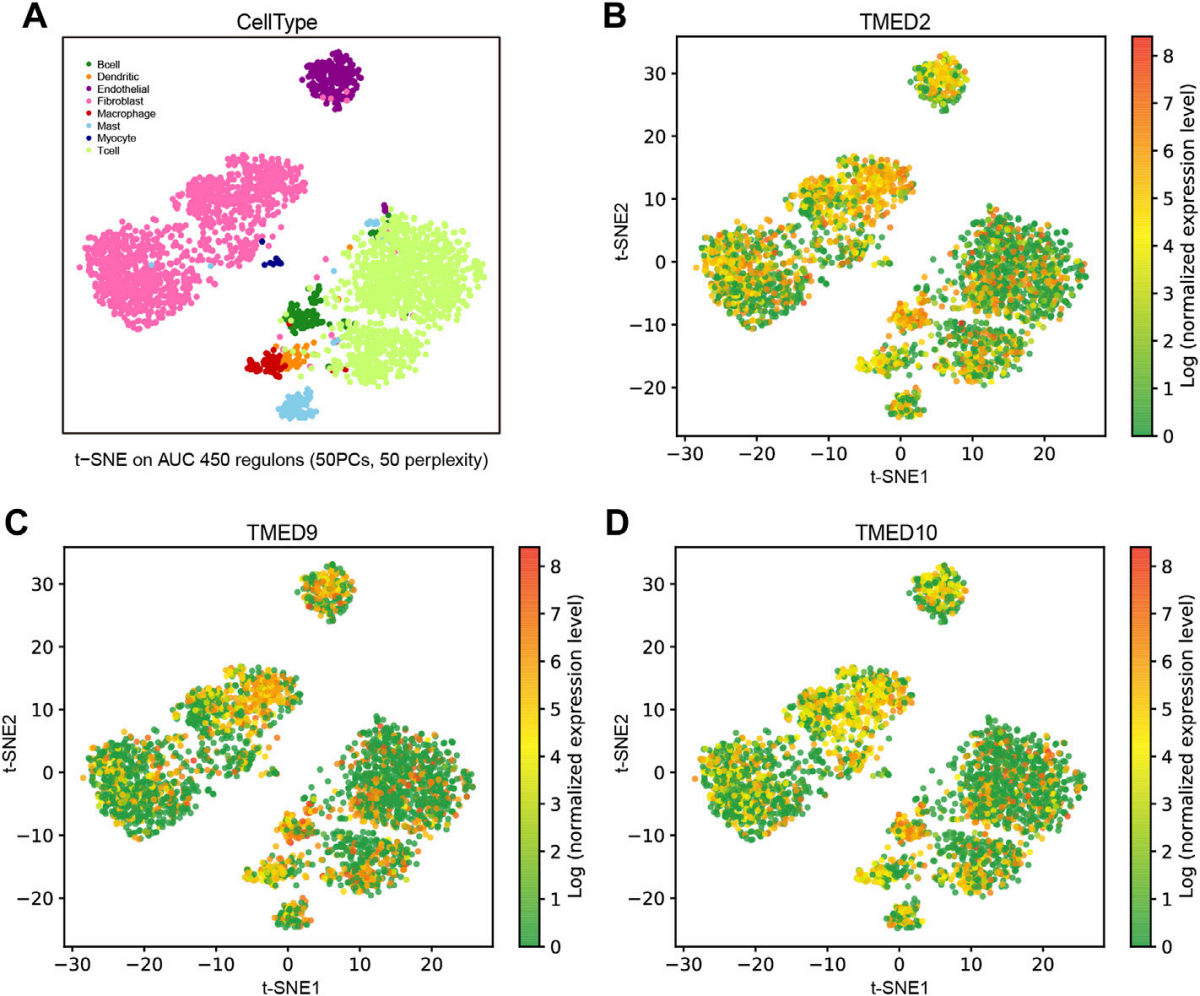
Expression analysis and single-cell analysis of genes TMED/2/9/10 in HNSC. **(A)** Distinguishing TMED2/9/10 enrichment and expression levels in different cell types of HNSC based on single-cell data. The t-SNE plots showed the expression levels in each cell of HNSC of **(B)** TMED2, **(C)** TMED9, and **(D)** TMED10.

To distinguish the enrichment and expression level of TMED2/9/10 in the different cell types of HNSC, a single-cell analysis was conducted by the GRNdb database. The t-SNE plots showed eight-cell types based on the HNSC single-cell dataset (Figure 7A). The expression levels of TMED2/9/10 were significantly increased in cancer-associated fibroblasts (CAFs), endothelial cells and B cells (Figures 7B–D).

### Correlation Between TMED2/9/10 Expression and Immune Cell Infiltration.

**FIGURE 8:**
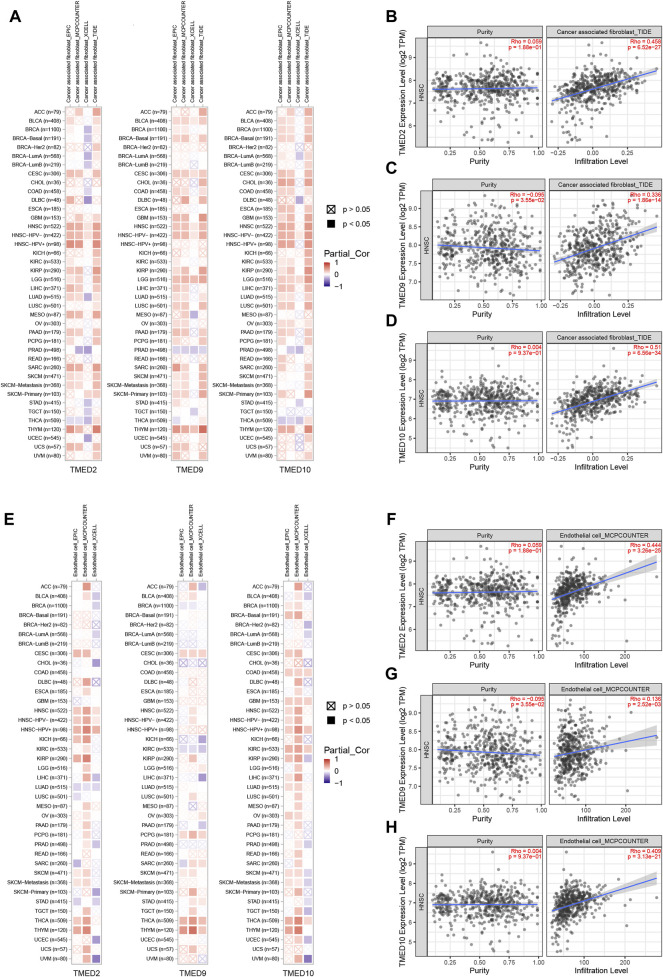
Correlation of TMED2/9/10 expression with immune infiltration levels in HNSC. **(A–D)** TMED2/9/10 expression was significantly positively related to infiltrating levels of cancer-associated fibroblast. **(E–H)** TMED2/9/10 expression had significant positive correlations with infiltrating levels of the endothelial cells.

To further explore the roles played by CAFs, endothelial cells and B cells in HNSC, we used TIMER 2.0 to investigate the association of TMED2/9/10 with various immune infiltrates in human cancers. The analysis showed that TMED2/9/10 were positively correlated with the level of immune infiltration of CAFs and endothelial cells in HNSC (Figures 8A–H). However, the multiple immune infiltration analysis results showed that TMED2/9/10 were not associated with the level of immune infiltration of B cells in HNSC (Supplementary Figure S2). So, we speculated that TMED2/9/10 might be involved in the immune infiltration process through CAFs and endothelial cells playing crucial roles in immune-oncology interactions.

### Potential Upstream Regulatory Factor Targets of TMED2/9/10 in HNSC

**FIGURE 9:**
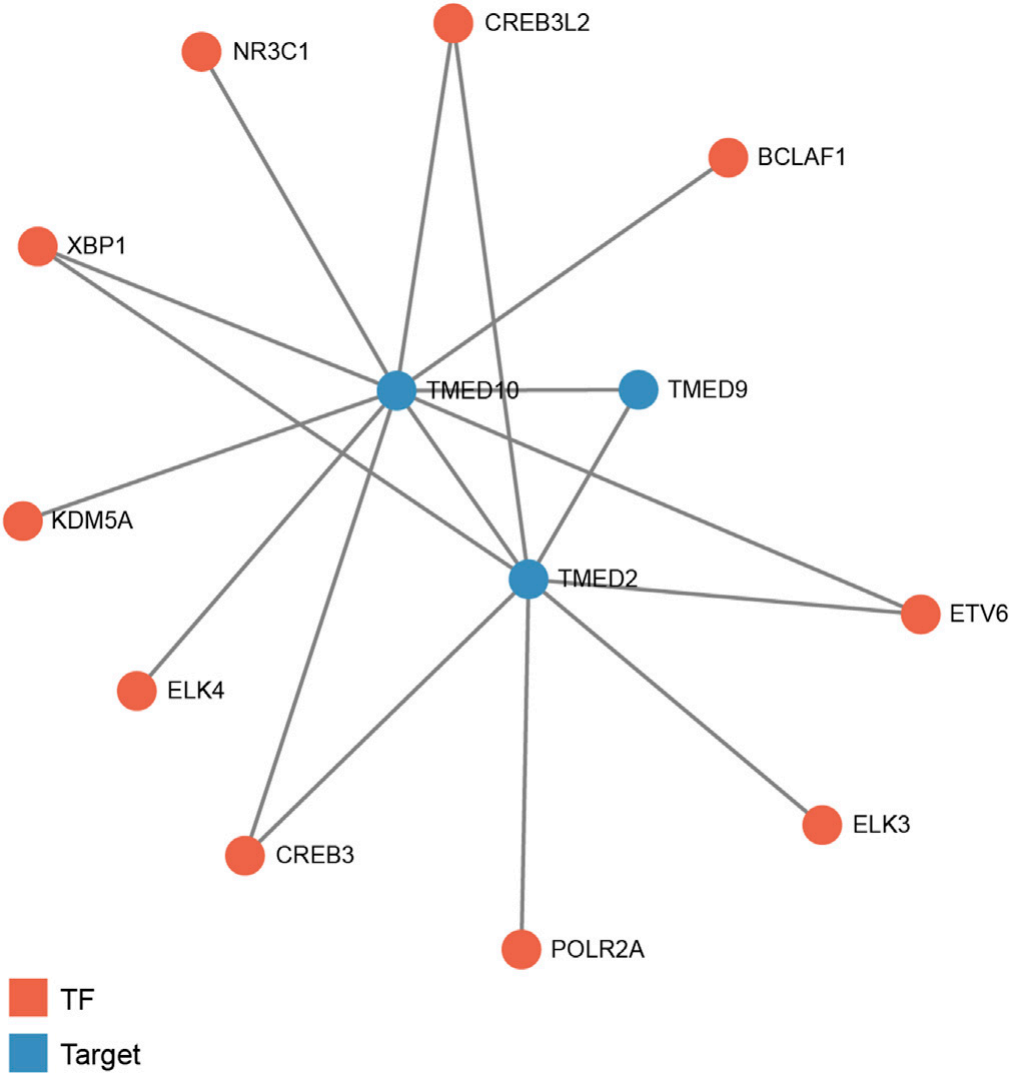
Predicted potential upstream regulatory transcription factors of TMED2/9/10 in HNSC based on Gene Regulatory Network database (GRNdb). TF: transcription factor.

To predict transcription factors that might play a regulatory role in the prognosis of HNSC, GRNdb was used to reveal the upstream regulatory transcription factors of TMED2/9/10. After the transcription factors-genes network analysis, we obtained transcription factors related to TMED2 and TMED10. The potential upstream transcriptional regulators predicted by TMED2 were XBP1, cAMP-responsive element-binding protein 3 (CREB3), cAMP-responsive element-binding protein three like 2 (CREB3L2), ETS transcription factor ELK3 (ELK3), ETS variant transcription factor 6 (ETV6), and RNA polymerase II subunit A (POLR2A). The potential upstream transcriptional regulators predicted by TMED10 were XBP1, CREB3, CREB3L2, ETS transcription factor ELK4 (ELK4), ETV6, nuclear receptor subfamily 3 group C member 1 (NR3C1), BCL2 associated transcription factor 1 (BCLAF1), and lysine demethylase 5A (KDM5A). Among the above regulators, the common transcriptional regulators were XBP1, CREB3, CREB3L2, and ETV6 (Figure 9).

### Analysis of TMED2/9/10 Through Correlation Heatmap and PPI Network

**FIGURE 10:**
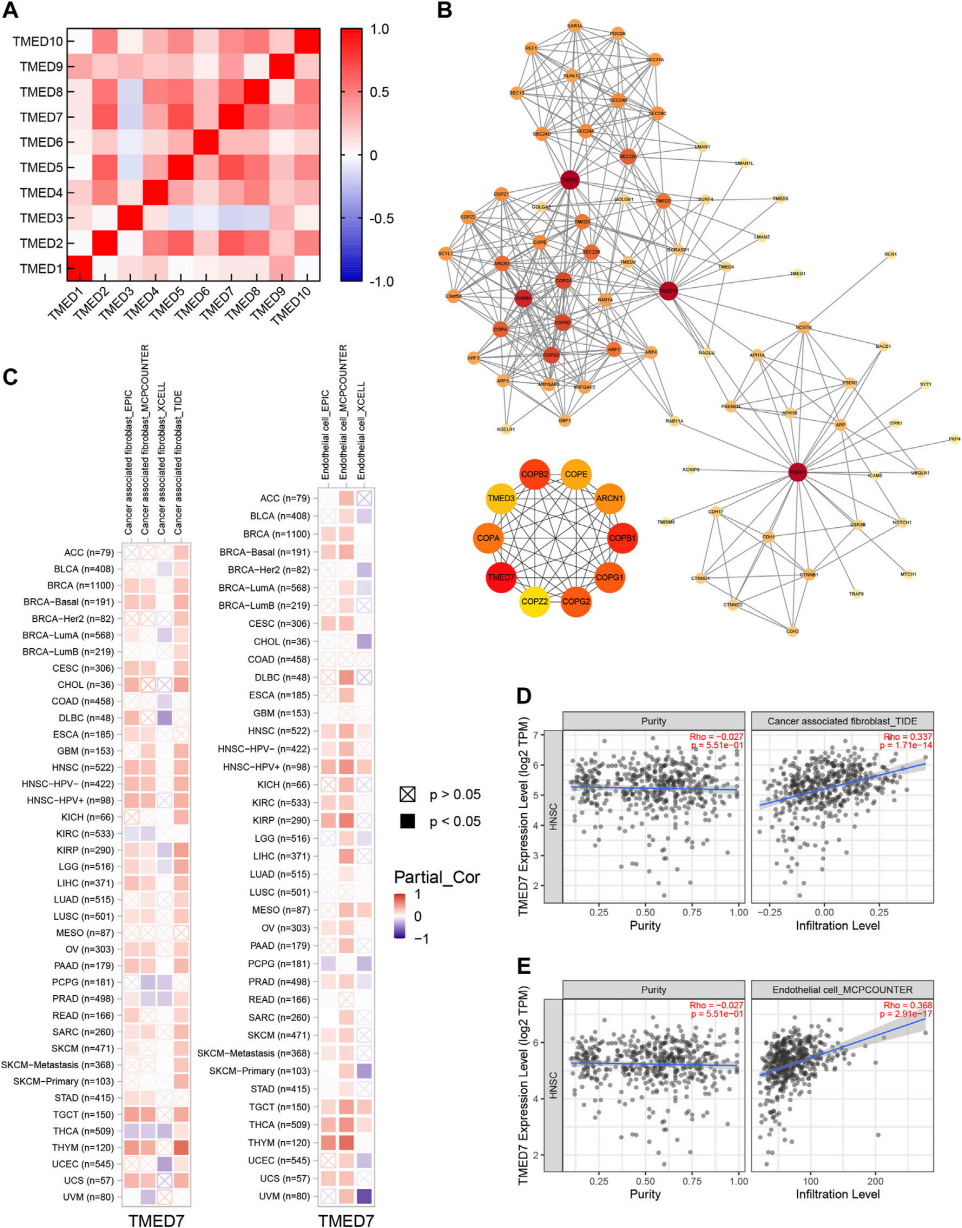
Co-expression and PPI of TMED genes in HNSC. **(A)** Heatmap of the TMED family proteins correlations in HNSC. **(B)** Protein-protein interaction network of TMED2/9/10, and the top 10 genes among them. **(C–E)** TMED7 expression had significant positive correlations with infiltrating levels of fibroblast and endothelial cells.

By constructing a correlation heatmap combining the TMED family in HNSC tissues, we found some positive correlations between TMED2/9/10. The results contributed to our insight into the prognostic impact of TMED2/9/10 versus HNSC patients (Figure 10A). The PPI network constructed by STRING showed genes having tight interactions with TMED2/9/10. By analyzing the association scores ranked by MCC method (Supplementary Table S1), we selected the ten highest-scoring hub genes: TMED7, COPI coat complex subunit beta 1 (COPB1), COPI coat complex subunit beta 2 (COPB2), COPI coat complex subunit gamma 2 (COPG2), COPI coat complex subunit gamma 1 (COPG1), coatomer protein subunit alpha (COPA), ARCN1, COPE, TMED3, and COPI coat complex subunit zeta 2 (COPZ2) (Figure 10B). By further exploring these 10 hub genes in immune infiltration using the TCGA-HNSC cohort in TIMER 2.0, we found that TMED7 expression levels showed a statistically significant positive correlation with CAFs and endothelial cells infiltration levels, which suggested that the hub gene TMED7 might play a role in the immune regulation of HNSC (Figures 10C–E).

## Discussion

Several studies have shown that the TMED proteins were involved in malignant tumors development. TMED2, as a critical factor in cell proliferation and differentiation, was found to exhibit cell-type-specific roles in cancer (Xiong et al., 2010; Shi-Peng et al., 2017). TMED3 was identified as a new prognostic biomarker because its expression was increased in the high-stage and -grade cohorts compared to the low-stage and -grade cohorts in renal cell carcinoma (Ha et al., 2019). Recent studies proposed the idea of TMED8 as a methylated gene regulating energy metabolism in neuroblastoma, which meant TMED8 could be used as a new target for therapy, drug development, and prediction of survival (Liu and Li, 2021). Also, highly expressed TMED9 significantly affected vascular invasion and poor prognosis in patients with hepatocellular carcinoma (Yang Y.-C. et al., 2021). Besides, it has been confirmed that isolated small peptides derived from the extracellular domain of TMED10 could treat cancers with abnormal TGF-β signaling activity by antagonizing TGF-β signaling (Nakano et al., 2017). However, the role of the TMEDs in HNSC has not been fully elucidated. To better explore the effect of the TMED family in HNSC, we picked out TMED2/9/10 for an in-depth study. We addressed the importance of TMED2/9/10 in HNSC from the perspectives of its expression in tumor tissues, prognostic value, expression-related genes, GO and KEGG enrichment analysis, single-cell analysis, and immune infiltration analysis, respectively.

In this study, we found that in HNSC tissues, the expression levels of TMED1/2/4/5/7/8/9/10 were significantly higher than those in normal tissues (Figure 1). In addition, we validated the expression levels of the TMED family in primary tumor and normal tissue in UALCAN (Figure 2). These results in UALCAN also showed us that the expression levels of TMED1/2/4/5/7/8/9/10 in patients were higher. Not only did the above results in TIMER and UALCAN prove the differential expression of the TMED family members, but also many studies explained the abnormalities of the TMED family in tumors. It was reported earlier that increased proliferation and invasion of ovarian cancer cells were positively correlated with ectopic expression of TMED2 (Shi-Peng et al., 2017). Because TMED3 was abnormally elevated in tumor samples from prostate cancer patients, it has also been identified as a potential drug target (Vainio et al., 2012). Evidence showed that the up-regulation of TMED5 in cervical cancer cells promoted malignant behavior and nuclear autophagy, affecting the progression of malignant tumors (Yang et al., 2019). Interestingly, elevated TMED2 and TMED9 expression levels in breast cancer patients were identified as poor prognostic factors (Lin et al., 2019; Ju et al., 2021). Therefore, the elevation of TMED proteins may significantly contribute to the proliferation and migration of cancer cells, thereby aggravating cancer progression. Furthermore, we performed survival curve analysis by GEPIA 2 and Kaplan-Meier Plotter successively to assess the clinical value of the TMED family. We first performed survival curve analysis of the TMED proteins with the GEPIA 2 database and found that TMED2/9/10 could be used as a prognostic marker for HNSC (Figures 3A–C). To ensure this inference, we performed a survival curve analysis in Kaplan-Meier Plotter for TMED2/9/10 (Figures 3D–F). The double-checked results indicated that highly expressed TMED2, TMED9, and TMED10 had a worse prognosis for patients with HNSC. In addition, we verified the diagnostic value of TMED2/9/10 in HNSC with the receiver operating characteristic curve. The result showed that the AUC values of TMED2/9/10 were greater than 0.5 (Figures 3G–I). Moreover, the combination of TMED2/9/10 held higher AUC values in the receiver operating characteristic curve (AUC = 0.847) (Figure 3J). Therefore, significantly elevated expression of TMED2, TMED9, and TMED10 in HNSC patients was considered a reliable diagnostic criterion. Meanwhile, the combination of TMED genes was a potential diagnostic biomarker in the future. To validate the result reliability, we compared TMED2/9/10 expression levels between normal tissues and HNSC tissues by using the GEO dataset as external validation. TMED2/9/10 were up-regulated in HNSC tissues than normal tissues (*p* < 0.001) (Figures 4A,B). Besides, we utilized the HPA database for IHC data to better validate our conclusions. The results indicated that the expression levels of TMED9 and TMED10 were significantly up-regulated in HNSC (Figures 4D,E), while there was no significant difference in TMED2 (Figure 4C). The above results suggested that the TMEDs might contribute to the development of HNSC.

TMED2/9/10 were significantly associated with critical clinicopathological features such as age, cancer grade, lymphatic metastasis, and cancer stage (Table 2). Thus, the result provided a new perspective on the relationship between clinicopathological features and prognosis. To better understand the function of TMED2/9/10 in HNSC, we first detected the mutation rates of TMED2/9/10 and found that the results were 4%, 7%, and 10%, respectively (Figure 5A). Hou et al. found an increased probability of non-alcoholic fatty liver disease in mice with heterozygous mutations in the TMED2 Hou et al. (2017). Therefore, we conjectured those TMED2/9/10 mutations might contribute to tumor development. Although TMED2/9/10 have higher mutation rates in HNSC, the relationship between them remains unclear, which deserves further exploration. To better explore the function of TMED2/9/10, we explored genes associated with TMED2/9/10 expression and studied their roles in the body. We excavated 5 genes most closely associated with TMED2/9/10 positive and negative, respectively, and found 52 genes co-expressed by TMED2/9/10 (Figures 5B–E). Afterward, we performed GO and KEGG analysis of the top thousand and co-expressed genes associated with TMED2/9/10 expression. GO enrichment analysis showed that the functions of TMED2/9/10 as well as co-expressed genes were mainly concentrated in the transferase complex, endoplasmic reticulum, intracellular protein transport cavity, cell-substrate, focal adhesion as well as coated vesicle (Figures 6A–D). KEGG enrichment analysis indicated that TMED2/9/10 and co-expressed genes were mainly involved in endocytosis, protein processing in the ER, focal adhesion pathway, focal adhesion, and phagosome (Figures 6E–H). The analysis results of these expression-related genes validated the function of TMED2/9/10. It has been demonstrated that during chorioallantois attachment, TMED2 functioned as a critical factor regulating the localization of fibronectin and vascular cell adhesion molecule 1 (VCAM1) (Hou and Jerome-Majewska, 2018). A study found that the cell biological mechanism of misfolded protein cargo entrapment was related to the targeting of TMED9 to the small molecule BRD4780 (Dvela-Levitt et al., 2019). In addition, membrane contact between the ER—Golgi intermediate compartment (ERGIC) and the ER-exit site (ERES) mediated by TMED9 constituted the occurrence of autophagosomes (Li et al., 2021). The transmembrane protein TMED10 was recently identified as a protein channel mediating vesicle translocation and secretion of termed cytosolic leaderless proteins (cytosolic proteins lacking a signal peptide) (Nguyen and Debnath, 2020; Zhang et al., 2020). TMED3, as an intracellular transporter, was knocked down to induce abnormalities in apoptosis-related proteins in lung squamous cell carcinoma (LUSC) cells. At the same time, TMED3 knockdown was involved in the regulation of LUSC cell function, for example, inhibition of proliferation, reduction of colony formation, induction of apoptosis and reduction of migration (Xie et al., 2021). These results suggest that TMED2/9/10 may cause the development or deterioration of HNSC by regulating vesicle trafficking or strengthening endocytosis.

In single-cell analysis, we first distinguished different cell types of the head and neck cancer ecosystem in Figure 7A. Interestingly, we found significantly higher expression levels of TMED2/9/10 in both CAFs, endothelial cells and B cells (Figures 7B–D). The results of the single-cell analysis of TMED2/9/10 implied its relationship with specific immune responses. Recently, it has been shown that TMED2 overexpression was negatively correlated with CD8^+^ T immune cell levels in HNSC, suggesting that TMED2 might initiate tumor development by altering the levels of immune infiltration in the tumor microenvironment (Sial et al., 2021). Also, Sun et al. found that TMED2 was required for cellular interferon (IFN) responses to viral DNA. MITA (mediator of IRF3 activation, also known as STING) had a vital role in the innate immune response to cytoplasmic viral dsDNA. Interestingly, TMED2 could bind to MITA, stabilize dimerization of MITA, and promote MITA translocation from the ribosome to the ER and the Golgi after viral infection. Moreover, the knockdown of TMED10 did not disrupt TMED2-mediated immune responses Sun et al. (2018). Therefore, we investigated whether TMED2/9/10 expression correlated with immune infiltration levels in HNSC. Our findings suggested that there was a strong positive relationship between TMED2/9/10 expression levels and infiltration levels of CAFs and endothelial cells (Figure 8), and TMED2/9/10 were not associated with the immune infiltration levels of B cells in HNSC (Supplementary Figure S2). According to previous studies, we knew that HNSC stroma was rich in infiltrating CAFs, with the highest concentrations accumulating near the invasive front of the tumor (Markwell and Weed, 2015). The adaptability of HNSC-CAF with myofibroblast characteristics led to the spread of extracapsular tumor cells, increased invasion, and lymph node metastasis (Marsh et al., 2011). At the same time, endothelial cells could vascularize the growing tumor mass and promote tumor cell invasion (Markwell and Weed, 2015). It has been found that after direct contact between endothelial cells and HNSC cells, the Notch ligand Jagged1 induced by mitogen-activated protein kinase (MAPK) in cancer cells activated the Notch signaling pathway in adjacent endothelial cells, ultimately promoting the formation of the capillary blastema (Zeng et al., 2005). In a word, microenvironmental rearrangements mediated by CAFs and endothelial cells have both direct and indirect effects on HNSC invasion. The high expression of TMED2/9/10 in immune cells validates the vital role of the TMED family in immunity.

The transcription factor-gene network showed the components closely related to TMED2/9/10 and HNSC (Figure 9). Among them, CREB3 was associated with the overall survival of HNSC patients and could be used as a prognostic biomarker for HNSC (Bornstein et al., 2016). Interestingly, our study found that the contribution of X-box binding protein-1 (XBP-1) to cancer provided new sights for this study. Abnormal accumulation of misfolded proteins in the endoplasmic reticulum (ER) led to ER stress. A compensatory mechanism called the unfolded protein response (UPR) was activated by cells responding to ER stress (Shajahan et al., 2009). XBP1 was an essential component of the UPR signaling pathway. XBP1 maintained proteostasis by stimulating the expression of chaperones and protein degradation machinery in the ER (Zhong et al., 2021). However, abnormal activity of XBP1 affected normal cell proliferation, apoptosis, metastasis, and ultimately tumorigenesis and tumor progression (Shi et al., 2019). Therefore, precise treatment against XBP1 may become a therapeutic direction for HNSC in the future.

We identified a positive correlation between TMED2, TMED9, and TMED10 (Figure 10A). Interestingly, genetic and biochemical experiments have shown that the stability of TMED proteins could be regulated by other family proteins: knockout or deletion of a TMED protein led to reduced or absent expression of TMED proteins from different subfamilies. For example, Denzel et al., when interrogating changes in the liver of mice heterozygous for the null mutation of TMED10, they found that the deletion of TMED10 not only resulted in developmental arrest before blastocyst formation but also decreased the expression of TMED9 and TMED3 proteins that interacted with them Denzel et al. (2000). So, it was evident that a complex network regulated the function of TMED2/9/10. To better combat HNSC, we should take advantage of the potential network of TMED2/9/10 at the same time. By using PPI network analysis, we identified hub gene TMED7 that was significantly associated with both TMED2/9/10 (Figure 10B). Similarly, we found strong positive correlations between infiltration levels of CAFs and endothelial cells and TMED7 expression in HNSC (Figures 10C–E). In particular, TMED7 could inhibit the Toll-like receptor 4 (TLR4) signaling pathway (Doyle et al., 2012). TLRs are important factors in the immune response, which can recognize invading pathogens and activate inflammatory responses. A previous study showed that TLR4 was aberrantly expressed in cancer cells, affecting the tumor microenvironment. To our surprise, there was evidence indicating that high expression of TLR4 was associated with poor prognosis in HNSC (Hu et al., 2021). Therefore, we hypothesized that activation of TMED7 could improve the prognosis of HNSC patients. In Supplementary Figure S1, we assessed the effect of the expression level of TMED7 on the prognosis of HNSC using the GEPIA 2 database. Although this result is not statistically significant, the trend of the survival curve is compatible with our inference. These pieces of evidence demonstrated that the hub gene TMED7 based on TMED2/9/10 could alter HNSC prognosis through immune infiltration. It reminds us that TMED2/9/10, as well as related genes, can be used as biological targets of HNSC.

Taken together, our results suggested that TMED2, TMED9, and TMED10 were significantly up-regulated in HNSC patients, and their upregulation was inversely correlated with HNSC prognosis. At the same time, we validated the above conclusions using GEO dataset and HPA database. Then, we used GO and KEGG enrichment analysis to elaborate in-depth on the functions of TMED2/9/10 and co-expressed genes. In addition, the results of the single-cell analysis and immune infiltration analysis also revealed that TMED2/9/10 affected the development of HNSC through immune cells. And the hub gene TMED7 and the transcription factor XBP1 were also expected to be potential prognostic markers and therapeutic targets for HNSC. So, we can infer that the transcription factor XBP1 might regulate the expression of TMED2/9/10, disturb their functions, boost immune cell infiltration, thereby promoting abnormal invasion of cancer cells and leading to poor prognosis of HNSC.

Regrettably, this study had some limitations. First, our selected sample data were confined to the TCGA and GEO databases, and further HNSC cohorts should be recruited in the future to confirm the results. Second, further experimental studies are required to validate the function of TMED2/9/10 at the cellular level. Finally, we still need to further explore the mechanism that TMED2/9/10 affect the prognosis of HNSC patients to provide more possibilities for clinical treatment.

## Conclusion

In conclusion, in this study, TMED2/9/10 and related genes entered our horizons as potential prognostic biomarkers, and the intersection of their functions helped researchers understand the pathogenesis of HNSC and provided a new approach for the treatment and prognosis of HNSC. At the same time, we analyzed the potential clinical value of the TMED family in the pathogenesis and development of HNSC and its associated oncogenic signaling pathways, providing clues for multi-target and TMED2/9/10-mediated targeted therapy. Finally, our in-depth exploration of TMED2/9/10 functions and immune infiltration allowed us better to understand the specifically expressed genes in HNSC patients, facilitating us to predict the survival of HNSC patients by the related genes. The above results supported targeting TMED2/9/10 as a new strategy for diagnosing and treating HNSC. However, the value of this conclusion for the prognosis of HNSC patients still needs further validation.

## Data Availability

The original contributions presented in the study are included in the article/Supplementary Material, further inquiries can be directed to the corresponding author.

## Supplementary Material

Supplementary Table S1 Top 10 in-network genes ranked by MCC method.

Supplementary Figure S1 The prognostic value of the TMED family in HNSC patients is based on the GEPIA 2 database. **(A)** TMED1; **(B)** TMED2; **(C)** TMED3; **(D)** TMED4; **(E)** TMED5; **(F)** TMED6; **(G)** TMED7; **(H)** TMED8; **(I)** TMED9; **(J)** TMED10.

Supplementary Figure S2 Correlation of TMED2/9/10 expression with immune infiltration levels of B cells in HNSC.
