# Supplementary material for: TMED2/9/10 Serve as Biomarkers for Poor Prognosis in Head and Neck Squamous Carcinoma
Source: Front Genet. 2022 Jun 8;13:895281. doi: 10.3389/fgene.2022.895281 (PMC9214264; doi:10.3389/fgene.2022.895281)
Supplement: Supplementary file 1 [file Table1.docx]

**Supplementary Table 1.** Top 10 in-network genes ranked by MCC method.

| Rank | Name | Score |
| --- | --- | --- |
| 1 | TMED7 | 5.19E+08 |
| 2 | COPB1 | 4.79E+08 |
| 3 | COPB2 | 4.79E+08 |
| 4 | COPG2 | 4.79E+08 |
| 4 | COPG1 | 4.79E+08 |
| 6 | COPA | 4.79E+08 |
| 7 | ARCN1 | 4.79E+08 |
| 8 | COPE | 4.79E+08 |
| 9 | TMED3 | 4.79E+08 |
| 10 | COPZ2 | 4.79E+08 |
